# How Exposure to Environmental Tobacco Smoke, Outdoor Air Pollutants, and Increased Pollen Burdens Influences the Incidence of Asthma

**DOI:** 10.1289/ehp.8380

**Published:** 2006-01-26

**Authors:** M. Ian Gilmour, Maritta S. Jaakkola, Stephanie J. London, Andre E. Nel, Christine A. Rogers

**Affiliations:** 1 U.S. Environmental Protection Agency, Research Triangle Park, North Carolina, USA; 2 Institute of Occupational and Environmental Medicine, University of Birmingham, Birmingham, United Kingdom; 3 National Institute of Environmental Health Sciences, National Institutes of Health, Department of Health and Human Services, Research Triangle Park, North Carolina, USA; 4 Department of Medicine, University of California Los Angeles, Los Angeles, California, USA; 5 Department of Environmental Health, Harvard School of Public Health, Boston, Massachusetts, USA

**Keywords:** air pollution, asthma, cigarette smoke, climate change, diesel exhaust, environment, inflammation, mechanisms, ozone, particulate matter, pollen

## Abstract

Asthma is a multifactorial airway disease that arises from a relatively common genetic background interphased with exposures to allergens and airborne irritants. The rapid rise in asthma over the past three decades in Western societies has been attributed to numerous diverse factors, including increased awareness of the disease, altered lifestyle and activity patterns, and ill-defined changes in environmental exposures. It is well accepted that persons with asthma are more sensitive than persons without asthma to air pollutants such as cigarette smoke, traffic emissions, and photochemical smog components. It has also been demonstrated that exposure to a mix of allergens and irritants can at times promote the development phase (induction) of the disease. Experimental evidence suggests that complex organic molecules from diesel exhaust may act as allergic adjuvants through the production of oxidative stress in airway cells. It also seems that climate change is increasing the abundance of aeroallergens such as pollen, which may result in greater incidence or severity of allergic diseases. In this review we illustrate how environmental tobacco smoke, outdoor air pollution, and climate change may act as environmental risk factors for the development of asthma and provide mechanistic explanations for how some of these effects can occur.

Allergic diseases are the sixth leading cause of chronic illness in the United States, affecting 17% of the population and costing the health care system about $18 billion annually [American Academy of Allergy, Asthma and Immunology (AAAAI) 2000]. Approximately 40 million Americans suffer from allergic rhinitis (hay fever), largely in response to common aeroallergens, resulting in 3.8 million lost days of work and school [Centers for Disease Control and Prevention (CDC) 2004]. Children with asthma are usually a subset of allergic individuals with more respiratory involvement characterized by chronic lung inflammation, airway hyperreactivity (AHR), and reversible airflow obstruction, whereas new asthma in adults may be more often non-allergic in nature [e.g., exercise-induced asthma (AAAAI 2000) and irritant-induced asthma (Tarlo 2003)]. Asthma commonly begins in childhood, but can also start in adulthood, and frequently requires doctor visits, long-term medication use, and in some cases hospitalizations. Currently, the CDC estimates the prevalence of asthma in the U.S. adult population to be 7.5% (16 million; CDC 2004).

Asthma and allergies have a strong hereditary and hence genetic component that likely works by modifying responses to ubiquitous environmental exposures. The factors affecting the onset of allergies and asthma are complex, and considerable attention has focused on the indoor environment [Institute of Medicine (IOM) 2000], as well as outdoor pollutant exposures (Peden 2003). Cigarette smoke and diesel exhaust particles (DEPs), in particular, have been shown to act synergistically with allergen exposure to enhance the severity of immune-mediated lung disease [California Environmental Protection Agency (California EPA) 1997; Diaz-Sanchez et al. 1997), and new evidence shows that ozone exposure and proximity to major roadways are associated with increased incidence of disease (Diaz-Sanchez et al. 2003; Heinrich and Wichmann 2004). In this review we illustrate how environmental tobacco smoke (ETS), outdoor air pollution, and climate change may act as environmental risk factors for the development of asthma and provide mechanistic explanations for how some of these effects can occur.

## Environmental Tobacco Smoke

Exposure to ETS—or passive smoking or exposure to secondhand smoke—is defined as exposure of a (nonsmoking) person to tobacco combustion products from smoking by others (Jaakkola and Jaakkola 1997). The fetus can be exposed either by the mother’s active smoking or by her exposure to ETS during pregnancy. The harmful substances of tobacco are then transferred across the placenta to the fetus.

Tobacco smoke contains more than 4,000 chemical substances, many of which are carcinogenic, mutagenic, irritating, or toxic. Exposure to ETS can be assessed by measuring air nicotine or respirable suspended particle concentrations with personal or stationary monitors (Jaakkola and Jaakkola 1997). Questionnaires are commonly used for assessing ETS exposure in health effects studies because they are relatively cheap and allow exposure assessment during different time periods and in different indoor environments. In addition, biomarkers can be measured as proxies for dose, including cotinine in body fluids and hair nicotine. Studies conducted in the United States and Europe have detected cotinine in urine as an indicator of passive smoking in > 80% of the nonsmoking populations (Pirkle et al. 1996; Riboli et al. 1990; reviewed by Jaakkola 2000). Questionnaire-based assessment of ETS exposure has varied from 7% in Finnish children (Jaakkola et al. 1994) to > 60% among Californian young adults (California EPA 1997).

We chose ETS as a model indoor pollutant in this review because at least some degree of exposure to ETS is very common worldwide, and studies have demonstrated that nonsmokers are exposed to concentrations high enough to be measured using biomarkers such as cotinine in body fluids. In addition, ETS contains several compounds that could plausibly cause health effects. For example, nicotine induces placental vasoconstriction, which leads to hypoxia of the fetus and consequently impaired maturation of the lungs. Irritant substances in ETS may induce chronic inflammation in the airways that could lead to a form of irritant-induced asthma (Tarlo 2003). In addition, animal studies and even some studies in children have suggested that in the presence of tobacco smoke exposure, hypersensitivity reactions to allergens are stronger. Indeed, based on the current knowledge, ETS is likely to be the most important indoor pollutant that is harmful for human health. The literature for this review on ETS and asthma is based on a Medline database (http://www.ncbi.nlm.nih.gov/entrez/query.fcgi?db=PubMed&itool=toolbar) search up to May 2004, but because of space constrictions, we reference previous reviews when possible and describe in detail only some recent longitudinal or incident case–control studies.

### ETS and induction of asthma in children.

Since the 1980s, numerous large studies have identified significant relations between parental smoking and development of asthma in children (Cook and Strachan 1997, 1998; Jaakkola 2000; J.J.K. Jaakkola and Jaakkola 2002). Meta-analyses based on these studies have shown a dose-dependent increase in children’s rates of asthma related to increasing number of household smokers (Cook and Strachan 1997), with the strongest effect detected in the youngest children (California EPA 1997; Strachan and Cook 1997, 1998). Maternal smoking has been reported to have a stronger effect than other household members’ smoking, raising concern of the potential role of prenatal tobacco smoke exposure. Recent studies have addressed the role of pre-natal versus postnatal exposure. A cohort study of 499 children of atopic parents from Boston found that maternal smoking during pregnancy was associated with an increased risk of asthma in the first year of life, with an odds ratio (OR) of 1.83 [95% confidence interval (CI), 1.12–3.00; Gold et al. 1999]. In a study based on the Finnish birth cohort that included almost 60,000 children, the risk of developing asthma among children 7 years of age increased in a dose-dependent pattern with the mothers’ smoking rates in pregnancy: OR 1.25 (95% CI, 1.09–1.44) for < 10 cigarettes/day and 1.36 (1.14–1.63) for > 10 cigarettes/day (Jaakkola and Gissler 2004). Significant associations persisted even after adjustment was made for low birth weight and duration of pregnancy, suggesting that the effect of passive smoking on asthma was independent of these factors.

### ETS and induction of asthma in adults.

Fewer studies have been published on ETS exposure and asthma in adults. To date one longitudinal, three case–control and at least six cross-sectional studies have reported associations between ETS exposure and adult-onset asthma, with ORs between 1.15 and 3.30 [reviewed by Jaakkola (2000) and by M.S. Jaakkola and Jaakkola (2002)]. In many studies the risk of asthma was more strongly related to workplace ETS exposure than to home exposure, and several studies showed evidence of a dose–response relation. A problem with most studies was that they included existing (prevalent) cases of asthma, making it impossible to distinguish development of asthma in adulthood from exacerbation of established disease. Another methodologic issue was the reliance on questionnaire reports of asthmatic symptoms or diagnosed asthma, as it may be difficult to distinguish between asthma and chronic obstructive pulmonary disease without clinical investigations. To address these challenges Jaakola et al. (2003) conducted a population-based case–control study with incident cases of asthma. The study assessed the effect of ETS exposure on adult-onset asthma and estimated the fraction of asthma attributable to ETS exposure in the past year. All new clinically diagnosed cases of asthma in individuals 21–63 years of age were recruited from 1997 to 2000 in a geographically defined area in southern Finland through all health care facilities diagnosing asthma in this area. A random sample of the source population (residents of the Pirkanmaa Hospital district, 21–63 years of age) was recruited as controls. A total of 521 asthma cases and 932 controls without asthma participated, including 239 persons with asthma and 487 controls who were never-smokers. These never-smokers formed the study population for the analysis on ETS exposure. Exposure to ETS was assessed on the basis of a self-administered questionnaire. Table 1 shows the results of ORs for asthma related to ETS exposure after adjusting for confounders including gender, age, education, parental atopy, and other occupational exposures in multivariate logistic regression. ETS exposure at work during the past 12 months was related to a significantly increased risk of adult-onset asthma, with an excess risk of 116% (95% CI, 26–272). Home exposure was associated with an even higher risk, but the CI range for this OR was wide because of a small number of study subjects being exposed at home (Table 1). Assessment of exposure quantitatively as cumulative exposure combining home and workplace exposures as cigarette-years suggested a dose–response relation with the risk of asthma. The fraction of exposed cases for which asthma was attributable to ETS exposure in the past year was 49% (95% CI, 16–69). The fraction of all asthma cases in the working age population (i.e., population-attributable fraction) attributable to ETS exposure in the last year was 8.0%.

### Conclusions on ETS and possibilities for prevention.

Considering the number of studies, their validity, evidence of dose–response relations, and biological plausibility, it can be concluded that *a*) there is some evidence for the effect of maternal smoking in pregnancy on the risk of asthma in childhood; *b*) post-natal exposure to ETS shows a causal link with the development of asthma in childhood; *c*) there is strong evidence that ETS is related to an increased risk of adult-onset asthma; and *d*) elimination or reduction of ETS exposure could prevent a considerable fraction of asthma in both children and adults.

Banning of smoking in the workplaces has been shown to reduce employees’ exposure to ETS and consequently fetal exposure of children of working pregnant mothers. For example, in Finland reformation of the tobacco control law in 1995 to protect employees from workplace ETS exposure led to a dramatic decline in tobacco smoke exposure at work in a 4-year follow-up of nine large- or medium-sized workplaces in southern Finland (Table 2; Heloma and Jaakkola 2003). This study also showed a decline in active smoking during the follow-up, suggesting that the legislation was able to affect home ETS exposure as well.

To reduce childhood exposure, parents should be educated and encouraged not to smoke, or if they already are smokers, to quit, through support in family planning and pre-natal health care and at schools. Based on studies from Finland (Jaakkola et al. 1994, 2001) and elsewhere (Owen et al. 1998), intensive support should be provided for certain groups at high risk for smoking during pregnancy and after delivery, such as young, single mothers with limited education. It has been suggested that effective secondary prevention could take place during doctor visits due to child’s respiratory problems. The most important issue for future research is how to eliminate (or at least reduce) ETS exposure effectively both in childhood and adulthood.

## Ambient Air Pollution and Asthma Induction

Although extensive evidence shows that ambient air pollution exacerbates existing asthma, a link with the development of asthma is less well established. This is primarily because few prospective studies with extensive exposure data have been conducted. However, in the past few years, some limited data sets have emerged to support associations between air pollution and incidence of asthma. The ambient air pollutants studied have included particulate matter (PM), nitrogen dioxide (NO_2_), sulfur dioxide (SO_2_), and ozone (O_3_).

The epidemiologic data on whether ambient air pollution contributes to the incidence of asthma are from five studies—three in children and two in adults. Of the childhood studies, one was a birth cohort from the Netherlands, the Prevention and Incidence of Asthma and Mite Allergy (PIAMA) study (Brauer et al. 2002), in which > 4,000 children were enrolled at birth and followed up to 2 years of age. Traffic-related air pollution was assessed on the basis of geographic information system models that were validated by a network of 40 monitoring stations at which NO_2_, PM_2.5_ (PM with aerodynamic diameter ≤ 2.5 μm), and reflectance measures for “soot” were assessed at four 2-week periods throughout a calendar year. The outcomes were parental reports of several p12222henotypes relevant to asthma; doctor-diagnosed asthma, bronchitis, ENT (ear, nose, and throat) infections, serious colds, or flu; and symptoms of wheeze and dry night cough. Small but statistically significant associations [relative risk (RR), 1.11–1.20] were found between traffic-related air pollution and wheeze, doctor-diagnosed asthma, ENT infections, and serious colds or flu.

An international collaborative study involving the Netherlands, Germany, and Sweden was established to examine traffic-related air pollution and childhood asthma (Gehring et al. 2002). Preliminary results published for 1,756 infants in Germany with follow-up through 2 years of age showed significant associations between dry cough at night in the first year of life and three measures of pollution—NO_2_, PM_2.5_, and soot, with ORs of 1.32–1.40. The associations persisted but were weaker in the second year of life (ORs 1.16–1.24). No association was found with wheezing, respiratory infections, or bronchitis.

The Children’s Health Study (CHS) includes > 6,000 children enrolled in 1993 and 1996 in 12 communities throughout southern California and provides a maximum range in exposure to ambient ozone, particles, acids, and oxides of nitrogen (McConnell et al. 2002). This report included 3,535 children without asthma at baseline and at least 1 year of follow-up during which 259 new cases of asthma diagnosis were reported. Air pollution was defined in this analysis by whether the community in which the child lived was in the bottom or top half of exposure for a given pollutant. The communities divided the same way for PM, NO_2_, and acids, but the high- and low-ozone communities were different. When ozone levels were examined alone, there was no increased risk of asthma with high exposure, and the peak level was actually related to significantly diminished risk. When children who participated in sports activities were examined, those who played three or more sports had an increased risk of asthma (RR = 1.8; 95% CI, 1.2–2.8). When the sports variable was further stratified by high/low air pollution status, there was no difference for particles, NO_2_, or acids, but an effect of three or more sports was strongly seen in the high-ozone communities. It should be noted that this finding was based on taking a very small category and dividing it into two subsets; only 9 of the 29 cases were in the low-ozone communities (RR = 0.8), versus 20 in the high-ozone communities (RR = 3.3; 95% CI, 1.9–5.8).

The evidence on air pollution and asthma incidence in adults comes from a cohort study of Seventh-day Adventists in California, known as the ASHMOG study. Investigators analyzed air pollution exposure in residentially stable nonsmokers in 1977 and followed up in 1982 and 1992. Exposure was based on the nearest monitoring station to the work and home addresses. In the first study, Abbey et al. (1995) examined incident asthma (79 cases) in relation to PM and reported a relative risk of 1.30 (95% CI, 0.97–1.73) for 1,000 hr/year exposure to concentrations of PM_10_ (PM with aerodynamic diameter ≤ 10 μm) that exceeded 100 μm/m^3^. A later study on ozone that included 115 incident cases of asthma (McDonnell et al. 1999) reported an increased risk in men for a 27 ppb (interquartile range) increase in ozone (RR = 2.09; 95% CI, 1.03–4.16) but no association in women.

In summary the results from the five prospective studies support a modest increase in risk for air pollution in relation to phenotypes relevant to asthma. The three studies of subjects old enough to have a firm diagnosis of asthma share limitations of uncertainty about when asthma started. This is a caveat for any prospective study of chronic disease, however, where it is difficult to distinguish possible exacerbation of preclinical symptoms from incidence of new asthma. The children’s studies are still quite small in scope, and activity patterns such as number of outdoor sports may also reflect other unrealized confounders. Numerous large-scale initiatives [e.g., the National Children’s Study (NCS 2005)] designed to track disease incidence and severity from before birth and relate those effects to environmental exposures will be able to more clearly define risk factors for the development of diseases such as asthma.

## Role of Prooxidative DEP Chemicals in Airway Inflammation and Allergic Adjuvancy

Four key questions [National Research Council (NRC) 1998) regarding the adverse health effects of PM on asthma are *a*) What is the mechanism by which PM affects cardiorespiratory morbidity and mortality? *b*) What are the PM components responsible for adverse health effects? *c*) What are the types and sizes of PM that are most potent? and *d*) Who in the population is more prone to adverse health effects? The impact of PM on the genesis and exacerbation of asthma is a good disease model to probe these questions (Nel et al. 1998). Atopic asthma is a disease of allergic airway inflammation, which from an ambient particle perspective could be approached by studying how PM pollutants in combination with common aeroallergens lead to *a*) the genesis of asthma, *b*) T-helper 2 (T_H_2) immune deviation by acting as adjuvants for allergic inflammation, and *c*) acute exacerbation of existing disease through an effect on AHR (Li et al. 2003a, 2003b).

To approach the questions listed above from an investigative perspective, reseachers have used DEPs as a model PM pollutant for *in vitro* and *in vivo* experiments (Nel et al. 1998). Several groups have demonstrated that DEPs can act as an adjuvant when combined with an experimental allergen, ovalbumin, resulting in enhanced IgE antibody production and increased allergic inflammation and AHR in mice (Miyabara et al. 1998; Whitekus et al. 2002; Xiao et al. 2003). To define the mechanistic basis for the proinflammatory effects of DEPs, Li et al. (2000, 2004) approached the problem from the premise that DEPs contain a large number of chemicals that play a role in inflammation. This includes transition metals as well as a host of organic chemical compounds. Among the organic chemicals, there is good evidence for the role of polycyclic aromatic hydrocarbons (PAHs) and quinones as toxicologically relevant compounds that give rise to reactive oxygen species (ROS) (Li et al. 2003a, 2003b; Monks et al. 1992; Penning et al. 1999; Xia et al. 2004). Silica gel chromatography was used to fractionate organic DEP extracts into aliphatic, aromatic, and polar compounds, which were enriched for *N*-alkenes, PAHs, and quinones, respectively (Alsberg et al. 1985; Li et al. 2000, 2004). Among these, the polar compounds are the most potent in participating in redox cycling reactions as determined by the interactions with the thiol derivative dithiothreitol (DTT). There is an excellent correlation between the results of the DTT assay and the ability of the various DEP fractions to induce oxidative stress in tissue culture macrophages and epithelial cells (Li et al. 2000, 2003b, 2004). Moreover, the same chemical groups are present in ETS, which may contribute to allergic airway inflammation in a fashion similar to DEPs.

**Figure 1:**
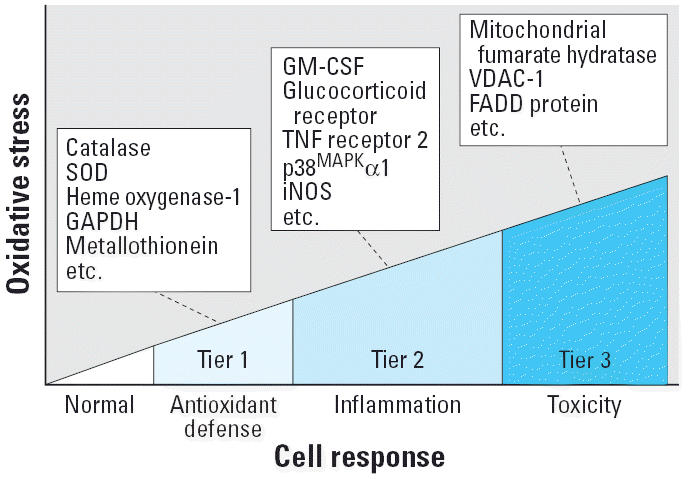
Hierarchical oxidative stress model in response to DEP exposure: proteome analysis of oxidative stress proteins shows a hierarchical response. Incremental doses of organic DEP extracts induce a series of incremental cellular responses that include increased antioxidant offense, inflammation, and cytotoxicity. At a lower level of oxidative stress (tier 1), antioxidant enzymes are induced to restore cellular redox homeostasis. At an intermediate level of oxidative stress (tier 2), newly expressed proteins often exhibit proinflammatory activity. At a high level of oxidative stress (tier 3), perturbation of the mitochondrial permeability transition pore and disruption of electron transfer result in cellular apoptosis or necrosis. Abbreviations: FADD, Fas-associating protein with death domain; GM-CSF, granulocyte-macrophage colony-stimulating factor; iNOS, inducible nitric oxide synthase; SOD, superoxide dismutase; TNF, tumor necrosis factor; VDAC-1, voltage-dependent anion channel 1.

Although ROS is generally accepted as playing a role in asthma, demonstration of this in animals and humans involves mostly indirect evidence from blood, bronchoalveolar lavage fluid, and exhaled air samples. Although direct measurement of ROS production in the respiratory tract is difficult, it is possible to use the biological impact of ROS generation in studying the effect of these radicals. When the production of ROS exceeds the ability of the affected tissue to neutralize the effects of the radicals, depletion in intracellular glutathione can result in a state of oxidative stress (Li et al. 2003a, 2003b). To develop new biomarkers for oxidative stress, several research groups used a proteomics display of newly induced oxidative stress proteins or posttranslationally modified proteins in PM target cell types such as macrophages and epithelial cells (Wang et al. 2005; Xiao et al. 2003, 2005) Upon exposure to prooxidative organic chemical components from DEPs, these cells show three tiers of oxidative stress, which are predictive of a possible *in vivo* hierarchical oxidative stress response (Figure 1) (Li et al. 2003a; Xiao et al. 2003). This model posits that as the level of oxidative stress increases, there is a transition from protective to injurious effects. Not only do these biological outcomes provide possible biomarkers of oxidative stress, but this model could also provide clues about susceptible human subjects.

In the first tier of oxidative stress, epithelial cells and macrophages respond by increasing expression of antioxidant and phase II enzymes (e.g., superoxide dismutase, catalase, glutathione peroxidase, glutathione reductase, γ-glutamate cysteine ligase, glutathione *S*-transferase, NADPH quinone oxidoreductase, heme oxygenase 1) via a genetic response pathway that acquires transcriptional activation of the antioxidant response element (ARE) by the transcription factor nuclear regulatory factor 2 (Nrf2) (Li et al. 2004). The Nrf2–ARE pathway protects against the injurious and proinflammatory effects of PM, and it has been proposed that a weakening of antioxidant defense (e.g., by the polymorphism of these genes) could determine which human subsets are susceptible to PM effects (Fryer et al. 2000). This notion is supported by recent data showing an increased asthma frequency in people with a polymorphism in the glutathione *S*-transferase who are more prone to increased IgE production in response to an intranasal co-challenge with allergen and DEPs (Gilliland et al. 2004). Similarly, there is evidence in animal asthma models that over-expression of heme oxygenase 1, a phase II enzyme with potent antioxidant effects, leads to a blunting of AHR and airway inflammation (Hisada et al. 2000).

In the second tier of oxidative stress, the activation of several intracellular signaling cascades could lead to transcriptional activation of proinflammatory genes (Xiao et al. 2003). Phosphoproteome analysis and anti-phosphopeptide immunoblotting demonstrate the activation of three major mitogen-activated protein (MAP) kinase cascades in epithelial cells and macrophages by DEPs, crude DEP extracts, and aromatic and polar DEP fractions (Wang et al. 2005; Xiao et al. 2003). Activation of these pathways leads to tumor necrosis factor-α, interleukin (IL)-8, IL-6, and vascular endothelial growth factor production that could be inhibited by MAP kinase inhibitors (Wang et al. 2005). It is noteworthy that the induction of AHR and airway inflammation can be blocked in a murine asthma model by employing a small-molecule inhibitor that interferes in the transcriptional activity of AP-1 (activator protein 1) proteins. There is good evidence that the above cytokines and chemokines affect airway inflammation and asthma, thereby contributing to the effects of the classical T_H_2 cytokines such as IL-4, IL-5, and IL-13. Nasal challenge studies with DEPs demonstrated that PM enhances T_H_2 cytokine production in atopic people during co-challenge with an allergen. These cytokine effects could form the basis for the adjuvant effects of DEPs on allergic inflammation in addition to the possibility that these particles affect the function of antigen presenting cells (Nel et al. 1998).

The third tier of oxidative stress involves mitochondrial perturbation, which can lead to apoptosis, apoptosis–necrosis, and superoxide generation (Hiura et al. 1999). In this regard, it is relevant that ambient ultrafine PM (PM < 0.1 μm) lodges in and induces structural damage in mitochondria (Li et al. 2003b). Although the clinical relevance of mitochondrial damage is uncertain, it is relevant that ultrafine PM collected in the Los Angeles basin has a higher content of redox-cycling organic compounds that are more prone to generate ROS in the DTT assay than does PM_2.5_ and PM_10_ (Li et al. 2003b). DEPs contribute significantly to the ultrafine PM load in an urban area. Although there is ample epidemiologic evidence that PM_10_ and PM_2.5_ play a role in asthma, there is a paucity of data on the role of ultrafine PM in asthma. However, on the basis of their small size, large surface area, high numbers, rich content of redox cycling chemicals, and high deposition efficiency, ultrafine PM may be particularly prone to induce airway inflammation and AHR (Penttinen et al. 2001). Since vehicular emissions have a significant output of ultrafine particles, it is relevant, therefore, that a number of studies have shown a link between traffic density and asthma exacerbation in inner city populations (Brauer et al. 2002).

In summary, there is growing evidence that PM-induced oxidative stress may be responsible for generating airway inflammation and AHR that are both markers and possibly precursors for the development of asthma. In addition, there is the realization that not all oxidative stress responses are injurious but also include a protective lower level of oxidative stress (tier 1 response; Figure 1) that could form the basis of disease susceptibility.

## Impact of Global Warming and Climate Change on Aeroallergens

### Climate and allergen load.

**Figure 2:**
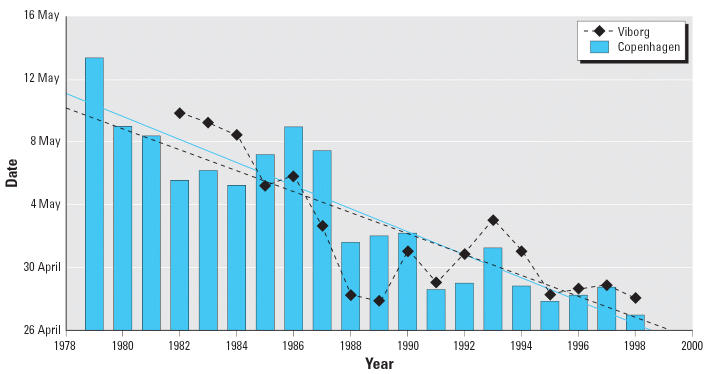
Peak date of airborne birch (*Betula*) pollen concentrations in Denmark, 1978–1999. Viborg: *Y*_m_ = 122.1; β = −0.67; *r* = −0.81; *p* < 0.001. Copenhagen: *Y*_m_ = 123.3; β = −0.74; *r* = −0.92; *p* < 0.001. Adapted from Rasmussen (2002).

It is generally thought that for atopic asthma or allergic disease to develop, both genetic predisposition and allergen exposure are required. Therefore, it is important to understand how impending climate change will affect the aeroallergens that elicit disease onset and precipitate symptoms. Climate warming that has occurred over recent decades (about 0.6°C thus far) has dramatically advanced budburst in spring (Fitter and Fitter 2002), therefore bringing forward the allergenic pollen season for spring-flowering taxa (Figure 2) (Rasmussen 2002; van Vliet et al. 2002). The rate of these advances (0.84–0.9 days/year) (Clot 2003; Frenguelli et al. 2002) provides some of the best evidence of the current impacts of recent climate change.

The most predictable climate changes over the next century involve an approximate doubling of atmospheric carbon dioxide (CO_2_) and a rise in average global temperature within the range of 1.4–5.8°C [Intergovernmental Panel on Climate Change (IPCC) 2001]. Numerous studies on plant responses to elevated CO_2_ indicate that plants will exhibit enhanced photosynthesis, biomass production, water use efficiency, and reproductive effort (Bazzaz 1990; Drake et al. 1997; Jablonski et al. 2002; LaDeau and Clark 2001; Stiling et al. 2004). These are considered positive developments for agriculture, but for allergic individuals they could augur increased exposure to airborne pollen. Some studies have in fact shown increased pollen production under conditions of elevated CO_2_. Ragweed (*Ambrosia artemisiifolia*) is a weed of open disturbed ground that produces potent pollen allergens. In controlled environment experiments, plants grown at elevated CO_2_ had greater biomass and produced 61–90% more pollen (Wayne et al. 2002; Ziska and Caufield 2000). Temperature and CO_2_ can also have interactive effects on pollen production. In experiments simulating early spring release from dormancy, ragweed plants grew larger, had more inflorescences, and produced more pollen than did later cohorts. Early cohorts under high levels of CO_2_ produced the same amount of pollen as those under ambient CO_2_, but later cohorts at high CO_2_ differed by producing 55% more pollen than their ambient CO_2_ counterparts (Rogers et al. 2006). Long-term records at pollen-monitoring stations in Europe show increasing annual totals for other types, including hazel, birch, and grass (Frei 1998; Spieksma 1995). Finally, a likely result of climate change will be shifts in the distributions of taxa as some species will be able to take advantage of new conditions, whereas others will not (Ziska 2003). For example, droughts may create open habitat that ragweed can colonize and therefore expand the range of this invasive and highly allergenic species in Europe resulting in individuals being exposed to new allergens.

### CO_2_, climate, and molds.

There is little evidence of the effects of climate change on fungal growth and reproduction, although the implications for allergic disease are just as important. As it is for pollen, exposure to fungal spores is unequivocally associated with exacerbations of allergy and asthma (IOM 2000). Long-term field experiments with elevated CO_2_ show that some fungi in mycorrhizal associations with trees have enhanced growth and sporulation (Klironomos et al. 1997; Treseder et al. 2003; Wolf et al. 2003). Although more evidence is needed to establish the certainty of these effects for a wider range of fungi over a gradual increase in CO_2_ (Klironomos et al. 2005), plausible arguments can be made for the likelihood of increased fungal biomass (and resulting sporulation) under climate change scenarios that would include increases in both mycorrhizal fungi to facilitate enhanced plant growth and saprobic fungi to degrade the increased plant biomass generated.

Fungi are also an important factor in indoor exposures leading to allergic and asthmatic events (Jaakkola and Jaakkola 2004). Several studies have shown that home dampness is a significant predictor of respiratory symptoms (Bornehag et al. 2001; Dales et al. 1991; IOM 2004). In a warmer climate, increased and more widespread reliance on air conditioning will occur, and the inevitable mismanagement of building ventilation will likely result in more cases of inappropriate moisture conditions in buildings. Changes in precipitation regimes are also anticipated, with heavier downpours and more widespread flooding. Increased flooding in coastal areas is projected with increases in sea level. All of these scenarios indicate a higher likelihood of wet interior surfaces that are prone to fungal growth. Inequities are likely to occur as lower-income families are less able to cope with expensive remediation or flood insurance (if available).

In summary, projected changes in climate over the next century will influence plant and fungal reproductive responses and alter the timing, production, and distribution of aeroallergens. Increased allergen exposures as a result of global warming, in combination with pollutant exposures such as DEPs that can act synergistically to enhance the allergic response, could mean increased respiratory difficulties in the years ahead.

## Summary of Key Issues

**Figure 3:**
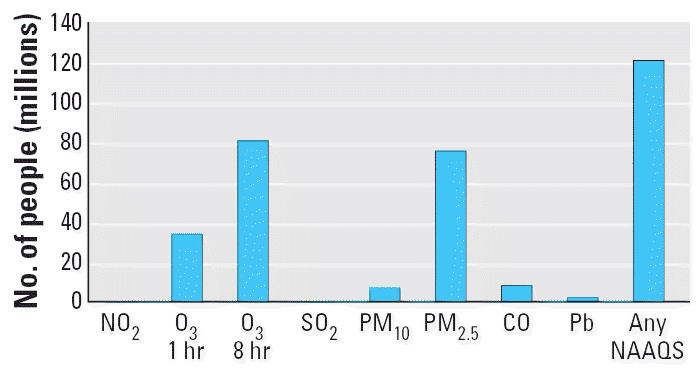
Number of people living in counties with air quality levels above the National Ambient Air Quality Standards (NAAQS) in 2000. Abbreviations: CO, carbon monoxide; Pb, lead. Data from U.S. EPA (2005).

The increasing asthma incidence worldwide is a complex issue that is not well understood. There are more cars and trucks on the road now than ever before, and half the U.S. population lives and works in areas out of compliance with U.S. EPA standards for ozone or PM_2.5_ (Figure 3). There is compelling evidence that exposure to ETS increases the risk of asthma in both children and adults, and although not conclusive, some prospective studies are showing small but statistically significant associations between air pollution and the incidence of asthma. In addition, increased temperature and CO_2_ due to climate change likely will result in increased production of pollen and fungal spores that could exacerbate symptoms of allergic diseases. More climatic and population studies linking chemical exposures, genetic susceptibility, daily activity patterns, dietary factors, and preexisting disease are needed to understand and quantify the associations between environmental factors and the development of asthma. In parallel, toxicologic studies can identify the role that particular chemicals such as diesel exhaust components and ozone play in the development and exacerbation of disease. In the short term such studies may help to determine how individuals can be protected, whereas in the long term they may provide useful information to establish limits for release of these chemicals to safe acceptable levels.

## Figures and Tables

**Table 1:** Exposure to ETS among cases and controls and adjusted OR of adult-onset asthma in relation to ETS exposure at home and at work.

ETS exposure	Cases [*n* (%)]	Controls [*n* (%)]	OR	95% CI
Past 12 months				
At work	34 (15.6)	41 (9.0)	2.16	1.26–3.72
At home	7 (3.0)	8 (1.7)	4.77	1.29–17.7
Cumulative cigarette-years (home and work)				
0	104 (43.5)	231 (47.4)	1.00	
1–49	26 (10.9)	91 (18.7)	0.80	0.48–1.36
50–99	22 (9.2)	44 (9.0)	1.30	0.71–2.35
100–149	19 (8.0)	25 (5.1)	2.01	1.02–3.99
≥ 150	68 (28.5)	96 (19.7)	1.84	1.21–2.80

**Table 2:** ETS exposure (%) in nine large- or medium-sized workplaces before and after 1995 reformation of the national tobacco control legislation in Finland.

Daily ETS exposure at work	1994–1995 (*n* = 605)	1995–1996 (*n* = 681)	1998 (*n* = 474)
Not at all	20.7	54.2	70.7
< 1 hr	28.8	28.6	17.5
1–4 hr	17.7	9.0	8.4
> 4 hr	32.9	8.2	3.4

Adapted from Heloma and Jaakkola (2003).
